# The Developmental Phenotype of the Great Toe in Fibrodysplasia Ossificans Progressiva

**DOI:** 10.3389/fcell.2020.612853

**Published:** 2020-12-08

**Authors:** O. Will Towler, Frederick S. Kaplan, Eileen M. Shore

**Affiliations:** ^1^Department of Orthopaedic Surgery, University of Pennsylvania, Philadelphia, PA, United States; ^2^The Center for Research in FOP & Related Disorders, Perelman School of Medicine, University of Pennsylvania, Pennsylvania, PA, United States; ^3^Department of Medicine, Perelman School of Medicine, University of Pennsylvania, Philadelphia, PA, United States; ^4^Department of Genetics, Perelman School of Medicine, University of Pennsylvania, Philadelphia, PA, United States

**Keywords:** fibrodysplasia ossificans progressiva, FOP, great toe malformation, hallux valgus, skeletal development, *ACVR1*, BMP signaling

## Abstract

Fibrodysplasia ossificans progressiva (FOP) is a rare genetic disorder in which extensive heterotopic ossification (HO) begins to form during early childhood and progresses throughout life. Although HO does not occur during embryonic development, children who carry the *ACVR1*^R206H^ mutation that causes most cases of FOP characteristically exhibit malformation of their great toes at birth, indicating that the mutation acts during embryonic development to alter skeletal formation. Despite the high prevalence of the great toe malformation in the FOP population, it has received relatively little attention due to its clinically benign nature. In this study, we examined radiographs from a cohort of 41 FOP patients ranging from 2 months to 48 years of age to provide a detailed analysis of the developmental features, progression, and variability of the great toe malformation of FOP, which include absent skeletal structures, malformed epiphyses, ectopic ossification centers, malformed first metatarsals and phalangeal fusion.

## Introduction

Fibrodysplasia ossificans progressiva (FOP) is an ultra-rare genetic disorder in which extensive bone ectopically forms in soft connective tissues, such as skeletal muscle, in a process known as heterotopic ossification (HO) (Shore and Kaplan, 2010). The *ACVR1* gene mutation that causes FOP and HO also alters the normal development of the skeleton (Cohen et al., 1993; Harrison et al., 2005; Schaffer et al., 2005; Mishima et al., 2014; Pignolo et al., 2019). The most frequently occurring mutation (∼97%) among FOP patients is *ACVR1*^R206H^ (Shore et al., 2006; Kaplan et al., 2008). This and other *ACVR1* mutations associated with FOP enhance signaling from this bone morphogenetic protein (BMP) type I receptor to increase activation of the downstream BMP signaling pathway (Kaplan et al., 2008; Shen et al., 2009; Allen et al., 2019).

The congenital skeletal malformation most commonly associated with FOP affects the first digit of the foot (also called the great toe or hallux), with this toe angled inward (hallux valgus) (Kaplan et al., 2008). Previous reports identified reduced first digit length, altered first metatarsal morphology, and distal phalangeal coalition (fusion) in multiple post-axial digits (i.e., digits 2–5) in patients diagnosed with FOP (Schroeder and Zasloff, 1980; Harrison et al., 2005); however, these studies examined only small cohorts (16 and 15 patients, respectively). While other case report series have been conducted, none have focused extensively on the forefoot malformations (Rosenstirn, 1918; Cohen et al., 1993). To investigate the frequency and types of malformations in all the digits of the foot, we conducted a detailed analysis of radiographs from 41 FOP patients with the *ACVR1*^R206H^ mutation.

## Materials and Methods

In this retrospective analysis, we reviewed radiographic images of the forefeet of 44 individuals with classic FOP. All individuals were established patients of one of the authors (FSK). Inclusion criteria were as follows: a clinical diagnosis of FOP made by the presence of congenital malformations of the great toes and by progressive HO in characteristic anatomic patterns; confirmation of the diagnosis by molecular genetic analysis that identified the presence of the recurrent *ACVR1* c617G>A;R206H FOP mutation (Shore et al., 2006); plain anterior-posterior (A-P) radiographs of the feet that had been obtained as part of routine clinical care. Exclusion criteria were the following: uncertainty of the patient’s age at the time the radiograph was acquired (2 subjects); and overexposure, underexposure, or degradation of the radiograph to the point analysis of the forefoot could not reasonably be completed (1 subject). This study was non-interventional and all patient data were deidentified prior to analyses through approval by the Institutional Review Board at the University of Pennsylvania.

Eight subjects (4M, 4F), including unaffected family members, served as age-matched controls for the purposes of comparing FOP-affected forefeet to normal development. These controls were not used for any statistical analyses. For cases without age-matched controls, radiographs were compared against anatomical sketches of foot development and clinical descriptions (Sarrafian, 2011). Patients with radiographs from multiple ages were not double-counted for any analyses. For any given feature that was absent at one age but present at another (e.g., ectopic ossification centers), patients were counted as having that feature.

**FIGURE 1:**
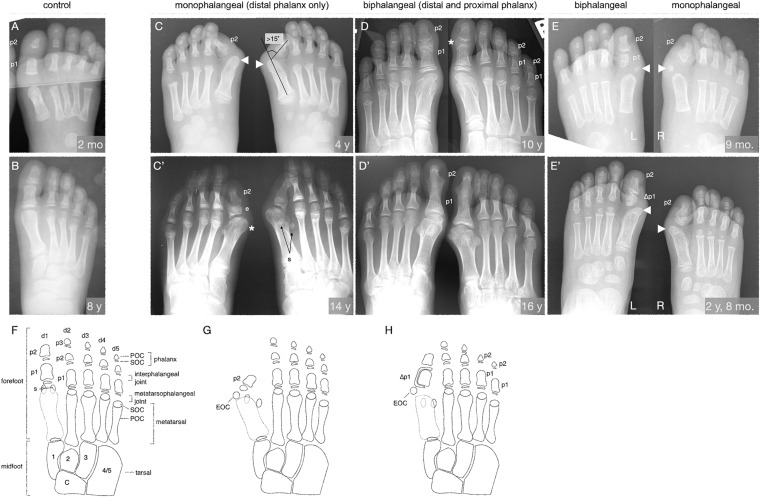
The FOP great toe malformation with monophalangeal or biphalangeal hallux. **(A,B)** Representative control radiographs at 2 months and 8 years of age. Note the presence of three phalanges (p) in each digit except the first, which has two. **(C–E)** Radiographs from three FOP subjects at different ages illustrate the two major presentations of the great toe malformation: monophalangeal hallux (p2 only; **C**,**E**) and biphalangeal hallux (p1 and p2; **D**,**E**). **(C)** Radiograph of a subject at 4 years of age shows bilateral monophalangism, with p1 being absent in both feet. Severe hallux valgus is evident (hallux valgus angle > 15°, illustrated). By this age, ectopic ossification centers (EOC; arrowheads) have fused to the metatarsal heads. **(C’)** At age 14, the same subject shows large epiphyses (e) associated with the remaining phalanx, medially deviated metatarsals with malformed heads (asterisk), and laterally deviated hallucal sesamoids (s); the fibular sesamoid is clearly visible, whereas the tibial sesamoid is masked by the metatarsal. **(D)** Radiographs of a subject at 10 years of age showing biphalangism, with both phalanges of the first digit present in both feet. Hallux valgus is minor, consistent with other subjects with this morphological progression. This subject additionally presents with biphalangeal digits 4 and 5, though this is not considered a hallmark of FOP. **(D’)** At age 16, the phalanges of the first digit have completely fused, which is characteristic of biphalangeal hallux in FOP (detailed in Supplementary Figure 1). **(E)** One subject, imaged at 9 months of age, presents with both a proximal and distal phalanx in the left foot and only the distal phalanx in the right foot. Note the asymmetric, amorphous shape of the proximal phalanx as contrasted with the rectangular, symmetrical morphology of the proximal phalanges in A. The right foot shows more severe hallux valgus than the left, but both feet have EOCs distal and medial to the first metatarsal (arrowheads). **(E’)** Two years later, the EOCs have both fused to the metatarsal head. **(F–H)** Illustrations of the skeletal elements of the human mid- and forefoot with **(F)** all usual elements in adulthood (control), **(G)** the monophalangeal FOP phenotype, and **(H)** the biphalangeal FOP phenotype. Digits (d) are numbered d1 to d5 from medial to lateral and phalanges (p) are numbered proximal to distal. By X-ray, primary ossification centers (POC) of each element are visible before their respective secondary ossification centers (SOC) can be seen. Midfoot elements, including the tarsals, are included for reference and are numbered according to their respective articulating digits. Sesamoids (s) are normally associated only with the hallux but may arise at the metatarsophalangeal joints of the second and/or fifth digits. The characteristic EOC medial and distal to the metatarsal of the hallux seen in FOP patients is marked in **(G,H)**. In **(E’,H)**, phalanges affected by LEPB or “delta phalanx” (also see Figure 2) are labeled as Δp1.

The timing, presence, and morphology of skeletal elements of the forefoot (metatarsals and phalanges) were compared against radiographs of control subjects and anatomical texts (Sarrafian, 2011). Phalanges were counted as dysmorphic if we observed clear deviations from the normal, rectangular “chess piece” morphology of all proximal and medial phalanges, or from the triangular morphology of the distal phalanges. Metatarsals were evaluated to be dysmorphic primarily on the basis of the shape of the metatarsal head, which should be smooth, rounded, and approximately symmetrical on its primary axis. Secondary ossification centers were considered dysmorphic if they were more cuboidal or hourglass-like in shape as opposed to the typical curved, linear shape (Figures 1A,B,F). Ectopic ossification refers to the appearance of superfluous radio-positive tissue either independent of or associated with the normal skeletal elements. Phalanges were counted as having coalition (AKA symphalangism) if no clear boundary line could be established between two phalanges within a single digit. The clinical definition of hallux valgus is that of an angle between the long axes of the metatarsal and proximal phalanx of the first digit greater than 15°. Hallucal sesamoids were counted as deviated if more than 50% of the fibular (lateral) sesamoid was visible beyond the lateral edge of the metatarsal.

Statistical analyses were performed using Prism 8 (GraphPad).

## Results

Among available FOP case files, the common FOP *ACVR1*^R206H^ mutation was documented in 41 subjects (22 M, 19 F; aged 0–48 years) with clinically diagnosed FOP and forefoot radiographs.

### Digit 1, Primary Ossification Centers

During digit development, ossification centers form and give rise to the individual skeletal elements (metatarsals and phalanges) of the digits (Sarrafian, 2011). The timing of the emergence and the final number of ossification centers and phalanges are the most general means by which to assess skeletal development in the digits of the foot. The expected numbers of phalanges in human feet is 2-3-3-3-3, counting from digits 1 to 5, with the elements of digit 1 being particularly broad (Figures 1A,B,F). Distal phalanges are distinguished by their comparatively triangular shape relative to the rectangular shapes of medial and proximal phalanges. Each phalanx consists of a primary ossification center (POC) connected proximally to a single secondary ossification center (SOC) via a growth plate (Sarrafian, 2011). The first digit metatarsal has only a proximal SOC, whereas each metatarsal of digits 2–5 has only a distal growth plate and SOC (Sarrafian, 2011). SOCs typically emerge as small, circular, radio-positive regions that expand into thickened, curved lines over time before gradually fusing with their associated skeletal element.

Examination of digit 1 in our 41 subjects provided an improved picture of the prevalence of characteristics that were previously recognized in FOP. These features (summarized in Table 1) included nearly fully penetrant hallux valgus (38/41; Figure 1C), metatarsal malformation (41/41; Figure 1C’), loss of the interphalangeal joint associated with loss of the proximal phalanx (21/41; Figures 1C,E), and the presence of an ectopic ossification center (EOC) medio-distal to the head of the first metatarsal (38/41; Figures 1E, 3).

**TABLE 1:** Major abnormal features of the first digit in FOP patients.

**Feature**	**Prevalence in subjects with FOP**	**Prevalence in general population**
Hallux valgus	38/41 (93%; 80.6–97.5%)	7.8% < 18 yo, 23% 18–65 yo (Nix et al., 2010)
Deviated hallucal sesamoids	13/21* (62%; 49.9–79.2%)	Data unavailable
Ectopic ossification centers	38/41 (93%; 80.6–97.5%)	Data unavailable
Monophalangeal hallux	21/41 (51%; 35.5–65.7%)	Unique to FOP
Lateral epiphyseal bracket, p1 of d1	16/21** (76%; 54.9–89.4%)	9 reported (Neil and Conacher, 1984; Low et al., 2013; Verma et al., 2014)

Incidence of various malformations in first digits of 41 analyzed subjects. Data are presented as fraction of total subjects, followed by percent and 95% confidence interval calculated using the Wilson/Brown test. *Of the 41 patients in the study, only 21 had fully visible hallucal sesamoids that could be reasonably assessed. **Only 21 of the 41 patients had a proximal phalanx of the first digit and therefore could be evaluated for this feature.

We additionally identified two distinct general pathologies for digit 1: one in which the proximal phalanx of digit 1 is absent (monophalangeal hallux; Figures 1C,C’,G), and the other in which this phalanx is present but malformed (biphalangeal hallux; Figures 1D,D’,H). Although the majority of radiographs of the feet showed similar bilateral malformations, one subject of the 41 examined had both phalanges in one foot, but only one in the other (Figures 1E,E’). Of the remaining 40 subjects, 20 were monophalangeal and 20 were biphalangeal for the first digit. Subjects lacking the proximal phalanx (monophalangism) generally showed much more severe hallux valgus, whereas those with both phalanges (biphalangism; p1 and p2, proximal and distal phalanges, respectively) had less severe hallux valgus. At age 15 and older, all subjects who formed both phalanges (biphalangeal) in the first digits (6/6; 95% CI, 61.0–100.0%) showed complete fusion of the two phalanges (Figure 1D’ and Supplementary Figure 1). No subjects with biphalangeal hallux showed such fusion before age 15 (0/15; 95% CI 0.0–20.4%). Malformation of the first metatarsal presented as an asymmetric or jagged metatarsal head and/or a broad diaphysis lacking the characteristic taper from each end to the midshaft (examples shown in Supplementary Figure 1).

### Digit 1, Secondary Ossification Centers

**FIGURE 2:**
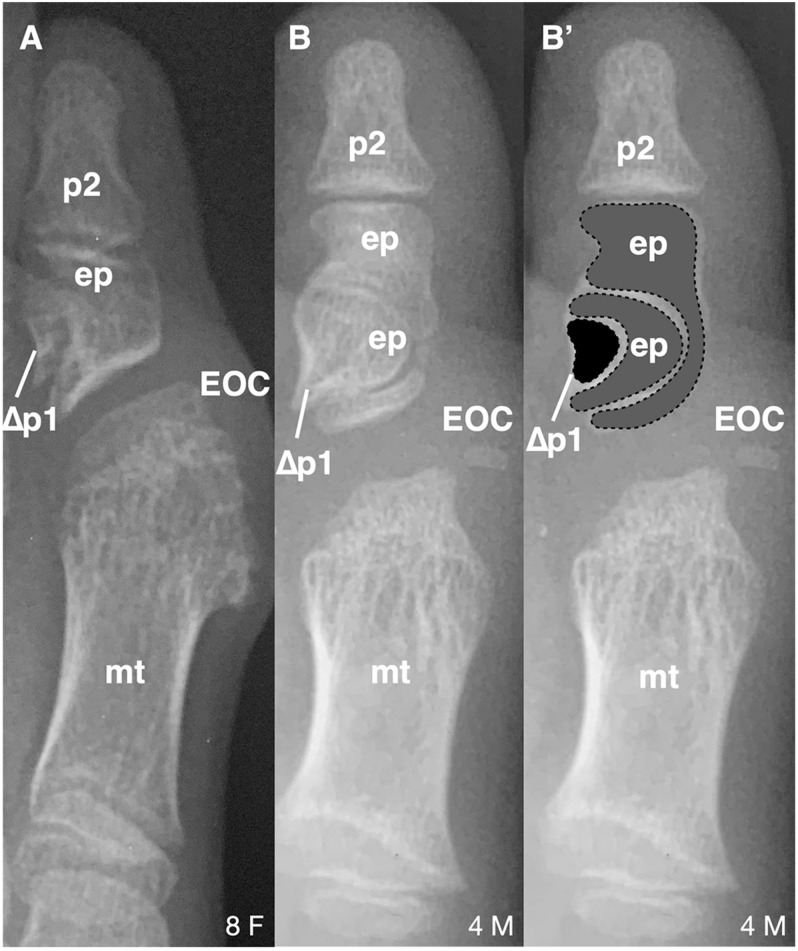
Longitudinal epiphyseal bracket in subjects with FOP. **(A)** Radiograph showing longitudinal epiphyseal bracket (LEPB; ep) of the proximal phalanx (p) of the great toe in a subject with FOP. A phalanx affected by LEPB may be referred to as delta phalanx, here labeled as Δp1. An apparent ectopic ossification center (EOC) distal to the metatarsal (mt) head is present. **(B)** Radiograph showing compound LEPB of the proximal phalanx, with outlines for clarity in **(B’)**. Dotted lines denote distinct osseous elements occurring in concentric hemi-circles. Age (in years) and sex (F, M) of each subject, bottom right of each panel.

Phalangeal SOCs emerge on average between 10 months and 2.3 years of age in digit 1, and between 2.5 and 4.4 years of age in digits 2–5, with males lagging behind females by about 6 to 12 months. SOCs first appear as small, circular, radio-positive regions that expand into thickened, and curved lines over time before gradually fusing with their associated skeletal element (Sarrafian, 2011). In both monophalangeal and biphalangeal cases of FOP, SOCs of first digit phalanges were frequently dysmorphic in a variety of presentations, including expansion on the proximal-distal axis (Figure 1C’), an hourglass-like shape with varying degrees of asymmetry (Figure 1D), and delta phalanx (Figure 1E’; also detailed below and in Figure 2).

**FIGURE 3:**
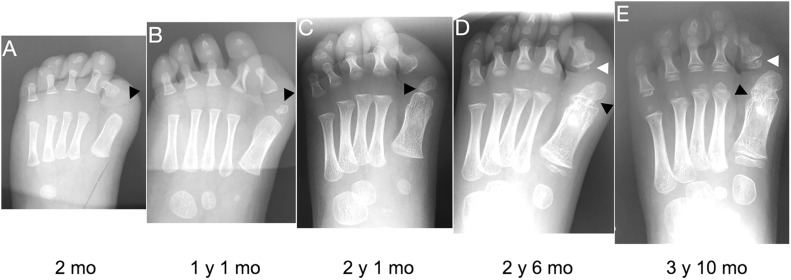
Progression of the FOP great toe malformation. Radiographs from a single FOP subject with monophalangeal hallux from birth to approximately 4 years of age illustrate the persistence of hallux valgus and the progression of the ectopic ossification center (EOC; black arrowheads). **(A)** At birth, the EOC is evident as a miniscule, radio-positive region distal and medial to the head of the first metatarsal. **(B,C)** Over time, the EOC increases in size and proximity to the metatarsal, with little to no growth distally relative to the phalanx. **(D)** The secondary ossification center of the remaining phalanx (white arrowheads **D,E**) forms immediately proximal to the phalanx, distinct from the EOC. **(E)** Finally, bone appears to fully bridge the EOC and the metatarsal (black arrowhead), fusing them together.

Of note, 16/21 (76%) subjects who have both first digit phalanges also exhibited some degree of longitudinal epiphyseal bracket (LEPB) of the proximal phalanx (Figure 2). LEPB is a rare skeletal malformation in which one epiphysis of a bone extends longitudinally along the diaphysis and is continuous with the opposing epiphysis, often leading to mediolateral deviation of the associated anatomy (Choo and Mubarak, 2013). LEPB is extremely rare in the first digit, with only 9 cases reported outside of FOP (Neil and Conacher, 1984; Low et al., 2013; Verma et al., 2014). In 3 of the 21 FOP subjects (14%), we also identified a novel malformation in which a compound LEPB produces concentric, ossified hemi-circles (Figures 3B,B’).

Nearly all subjects (39/41; 95%) had an EOC distal and medial to the metatarsal head, as previously described (Harrison et al., 2005). In all seven subjects for whom radiographs at multiple ages were available, including one with a series of five radiographs (Figure 3), the EOC expanded and ultimately fused with the head of the first metatarsal. This fusion occurred regardless of the presence or absence of the proximal phalanx of digit 1 (Figure 1), suggesting the EOC is not a misaligned or vestigial phalanx.

### Digits 2–5, Primary and Secondary Ossification Centers

Previous radiographic studies of the post-axial digits (digits 2–5) of patients with FOP noted occasional distal interphalangeal fusion and absence of the fifth digit medial phalanx; however, such malformations are not uncommon in the general population (Ceynowa et al., 2018). To evaluate the effects of the FOP mutation on digits 2–5 of the feet, we assessed absence of phalanges, delayed appearance of epiphyses, and interphalangeal fusion (Table 2). Similar to observations of these features in the general population, phalanges of these digits were never absent in FOP except the middle phalanx of digit 5 (22%). Additionally, distal interphalangeal fusion occurred with increasing frequency from medial to lateral (digits 2–5) and epiphyses were either delayed or absent at increasing frequencies from medial to lateral. Based on reported studies, the frequencies of these digit variations in FOP are within the range of those within the general human population (Le Minor et al., 2016; Ceynowa et al., 2018).

**TABLE 2:** Frequency of anomalous radiographic features of the forefoot of individuals with FOP.

**Feature**	**d1**	**d2**	**d3**	**d4**	**d5**
Absent phalanx	51.2%* (36.5–65.7%)	0.0% (0–8.6%)	0.0% (0–8.6%)	0.0% (0–8.6%)	22.0% (12.0–36.7%)
Absent/delayed phalanx SOC	9.8% (3.9–22.5%)	19.5% (10.2–34.0%)	26.8% (15.7–41.9%)	29.3% (17.4–44.5%)	43.9% (29.9–59.0%)
Distal inter- phalangeal fusion	12.2% (5.3–25.5%)	2.4% (0.1–12.6%)	9.8% (3.7–21.6%)	14.6% (6.9–28.4%)	24.4% (13.8–39.3%)
Malformed metatarsal	100% (91.4–100.0%)	4.9% (0.9–16.1%)	4.9% (0.9–16.1%)	4.9% (0.9–16.1%)	4.9% (0.9–16.1%)

**FIGURE 4:**
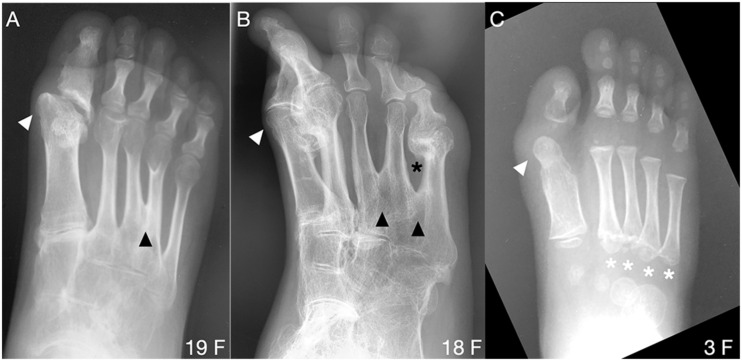
Uncommon forefoot phenotypes in FOP. **(A,B)** Radiographs from two patients reveal osseous syndactyly (black arrowheads) between metatarsals of digits 3 and 4 **(A)** and among digits 3, 4, and 5 **(B)**. White arrowheads indicate the dysmorphic metatarsal heads (all panels), corresponding to the position of the ectopic ossification center noted in nearly all subjects with FOP. In B, extra-articular HO bridges the metatarsophalangeal joint of digit 5 (black asterisk) and HO is present in d2. **(C)** One of two identified patients presenting with atypical proximal metatarsal growth plates in digits 2–5 (white asterisks). Age (in years) and sex (F, M) of each subject, bottom right of each panel.

Incidence of specific skeletal features in radiographs of the forefoot of FOP patients were based on comparisons to the expected features as described (Sarrafian, 2011). All percentages are based on the total cohort of 41 subjects. Because this study was cross-sectional, “absent” vs. “delayed” radiographic appearance of secondary ossification centers (SOC) cannot be distinguished and are thus grouped together; however, because the medial phalanges ossify extremely early in life when present, “absent” is a more certain diagnosis in that category. A digit was scored as having distal interphalangeal fusion if the rectangular morphology of the medial phalanx was both clearly present and continuous with the approximately triangular distal phalanx. All subjects showed a malformed first metatarsal with a dysmorphic head and/or broad diaphysis lacking the usual tapering from head to midshaft. Two subjects (4.9%) accounted for all other metatarsal malformations in digits 2–5 (detail, Figure 4). Data are presented as percentage of subjects and 95% confidence interval, calculated using the Wilson/Brown analysis. *The absent phalanx in digit 1 results in monophalangeal hallux. These data are also indicated in Table 1.

In contrast to metatarsals of the first digit, metatarsal malformations in digits 2–5 of subjects with the FOP *ACVR1*^R206H^ mutation were extremely rare; however, osseous syndactyly (fusion) is more common in rare cases of FOP with a mutation other than *ACVR1*^R206H^ (Kaplan et al., 2009; Gucev et al., 2019). Two of the 41 *ACVR1*^R206H^ subjects examined showed metatarsal syndactyly, one with fusion of digits 3 and 4 (Figure 4A), and the other with fusions between digits 3 and 4 as well as between digits 4 and 5 (Figure 4B). Additionally, the latter subject showed clear extra-articular fusion of the fifth metatarsophalangeal joint (Figure 4B). Although metatarsals normally have distal but not proximal secondary ossification centers, two FOP subjects presented with apparent proximal secondary ossification centers of all metatarsals in digits 2–5 (Figure 4C).

## Discussion

The great toe malformation of FOP has been an enigma since its first association with the disease (Rosenstirn, 1918) but has received little attention due to its benign nature relative to the extensive HO in FOP. Here, we examined the skeletal features of digit malformation in FOP patients with the *ACVR1*^R206H^ mutation to provide a more detailed description of this skeletal phenotype and thus better determine its etiology and potential implications for the effects of the mutation on other aspects of skeletal and joint biology. The findings presented here identified altered patterns of growth and morphology along the proximal-distal axis of the forefoot including aberrant skeletal elements and ectopic ossification centers consistent with the malformed, short great toes that are a characteristic and diagnostic clinical feature of FOP. We further determined that the great toe malformations result from two distinct phenotypes, monophalangeal and biphalangeal.

Although the radiographic evidence in our cross-sectional study is insufficient to understand the full consequences of the FOP ACVR1 mutation on the skeleton and joints, expression of the common ACVR1-R206H mutation in mouse models induces similar bone and joint malformations in the digits to those observed in patients (Chakkalakal et al., 2016). FOP mouse models have digit 1 joints that are fused and diminished from birth, supporting that ACVR1 is important for the appropriate specification of joint structures in digit 1 and that increased BMP pathway signaling by the ACVR1 mutations in FOP alter the skeletal elements.

Further evidence that ACVR1 is important in specifying joint structures stems from the ectopic ossification center (EOC) in digit 1 that is present in the majority of human subjects with FOP (Table 1). As seen in Figure 3, this EOC appears to expand toward and then fuse with the metatarsal head. This fusion appears visually consistent with normal growth plate closure in other digits of unaffected individuals; that is, a “seam” between the EOC and metatarsal head is evident from childhood to early adolescence (Figures 1D–E; also Figure 3E) that becomes difficult or impossible to detect in late adolescence to adulthood. This progression suggests that the EOC is an ectopic secondary ossification center (SOC) that is connected to the metatarsal head via an ectopic growth plate. Such a phenotype is consistent with the aberrant morphology and growth of endogenous SOCs in subjects with FOP. In the absence of MRI data, which could help differentiate among fibrocartilage, tendon, and epiphyseal cartilage, as well as the absence of additional radiographic viewing planes, we cannot rule out the possibility that the EOC is an ectopic sesamoid that eventually fuses to the metatarsal along its associated tendinous tissue; however, our assessment is that the data better support that the EOC is connected to the metatarsal head by a growth plate, and thus constitutes an ectopic SOC.

As mentioned above, one possible etiology for the malformed great toe in FOP comes from considering sesamoids, which are small, ossified structures that support the mechanical function of certain joints, particularly at the knees, and digits. The sesamoids found in human feet most frequently are the tibial and fibular sesamoids (collectively referred to as the hallucal sesamoids), which articulate with the ventral groove of the first metatarsal head in the metatarsophalangeal joint. Rarely, sesamoids also arise singly in other digits (Bizarro, 1921). Our data concerning digits 2 through 5 are unable to conclusively show whether sesamoid development in the FOP population deviates from that in the general population; however, hallucal sesamoids in FOP subjects were often laterally deviated (possibly as a result of the malformed metatarsal head), a condition that leads to unbalanced mechanical forces resulting in hallux valgus (Boike et al., 2011).

One previously unrecognized finding from this study is the two distinct presentations of the great toe phenotype, one monophalangeal and one biphalangeal. This might at first seem likely to be explained by background genetics of individual subjects; however, one subject having both presentations, one in either foot, suggests that instead, the FOP mutation, and its resulting increased BMP pathway signaling, broadly dysregulates the signaling pathways that determine the proximal-distal pattern of ossification in the first digit. The number and positions of ossification centers is then determined stochastically from those altered parameters. Indeed, digit patterning is not dependent on a prescribed number of skeletal elements, but instead on complex reaction-diffusion gradients that incorporate BMP pathway activity as a major factor, the disruption of which can have a variety of outcomes on digit and phalanx number and growth (Badugu et al., 2012; Raspopovic et al., 2014; Huang et al., 2016).

Each of the phenotypes that we have noted in FOP patient digits—growth plate placement and alignment, sesamoid alignment, and phalanx number—are components of the broader and coordinated processes of embryonic joint development (Grüneberg and Lee, 1973; Ray et al., 2015; Huang et al., 2016), and are consistent with findings of generalized joint dysplasia, malformation, and susceptibility to degenerative arthropathy in the FOP population (Towler et al., 2019). Although causal relationships among the phenotypes reported here cannot yet be conclusively established, these data provide a more comprehensive and detailed view of the developmental phenotype of the great toe in patients with FOP and provide new insight into the roles of *ACVR1* and BMP pathway signaling in human skeletal and joint development.

## Data Availability Statement

The raw data supporting the conclusions of this article will be made available by the authors, without undue reservation.

## Ethics Statement

The studies involving human participants were reviewed and approved by the Institutional Review Board of the University of Pennsylvania. Written informed consent from the participants’ legal guardian/next of kin was not required to participate in this study in accordance with the national legislation and the institutional requirements.

## Author Contributions

OT constructed the figures and tables. OT and FK collected and analyzed the data. OT and ES prepared the initial draft and final manuscript. All authors contributed to project development, manuscript composition, and editing.

## Conflict of Interest

The authors declare that the research was conducted in the absence of any commercial or financial relationships that could be construed as a potential conflict of interest.

## Supplementary Material

Supplementary Figure 1 Symphalangism of biphalangeal hallux in subjects with FOP. **(A–D)** Radiographs of four patients at various ages with biphalangeal hallux. **(A,B)** Subjects with both proximal and distal phalanges (p1, p2) appear to show separation of those phalanges throughout childhood and early adolescence. The phalanges are closely juxtaposed prior to age 15 and may already be fused (higher magnification, below), but show a clear demarcation between the two skeletal elements (arrowheads). **(C,D)** At ages 15 and older, proximal and distal phalanges are fused. The distinct shapes of the proximal extremities of the phalanges (asterisks) suggest that both phalanges were initially present although could not be verified in subjects lacking radiographs from younger ages. **(E)** An unaffected patient with clear separation of the proximal and distal phalanges of digit 1. ep, epiphysis; gp, growth plate; LEPB, lateral epiphyseal bracket; mt, metatarsal. Age (in years) and sex (F, M) of each subject, bottom right of each panel.

## References

[B1] Allen R. S. Tajer B. Shore E. M. Mullins M. C. (2019). FOP-ACVR1 signals by multiple modalities in the developing zebrafish. *Elife* 9:e53761. 10.7554/eLife.53761 PMC7478894 32897189

[B2] Badugu A. Kraemer C. Germann P. Menshykau D. Iber D. (2012). Digit patterning during limb development as a result of the BMP-receptor interaction. *Sci. Rep.* 2:991. 10.1038/srep00991 23251777 PMC3524521

[B3] Bizarro A. H. (1921). On sesamoid and supernumerary bones of the limbs. *J. Anat.* 55 (Pt 4), 256–268. 17103926 PMC1262938

[B4] Boike A. Schnirring-Judge M. McMillin S. (2011). Sesamoid disorders of the first metatarsophalangeal joint. *Clin. Podiatric Med. Surg.* 28 269–285. 10.1016/j.cpm.2011.03.006 21669339

[B5] Ceynowa M. Rocławski M. Pankowski R. Mazurek T. (2018). The prevalence and ossification pattern of the biphalangeal and triphalangeal lateral toes. *Surg. Radiol. Anatomy* 40 1039–1045. 10.1007/s00276-018-2027-z 29667031 PMC6132864

[B6] Chakkalakal S. A. Uchibe K. Convente M. R. Zhang D. Economides A. N. Kaplan F. S. (2016). Palovarotene inhibits heterotopic ossification and maintains limb mobility and growth in mice with the human ACVR1(R206H) Fibrodysplasia Ossificans Progressiva (FOP) mutation. *J. Bone Miner. Res.* 31 1666–1675. 10.1002/jbmr.2820 26896819 PMC4992469

[B7] Choo A. D. Mubarak S. J. (2013). Longitudinal epiphyseal bracket. *J. Children’s Orthopaed.* 7 449–454. 10.1007/s11832-013-0544-1 24432108 PMC3886355

[B8] Cohen R. B. Hahn G. V. Tabas J. A. Peeper J. Levitz C. L. Sando A. (1993). The natural history of heterotopic ossification in patients who have fibrodysplasia ossificans progressiva. a study of forty-four patients. *J. Bone Joint Surg. Am.* 75 215–219. 10.2106/00004623-199302000-00008 8423182

[B9] Grüneberg H. Lee A. J. (1973). The anatomy and development of brachypodism in the mouse. *J. Embryol. Exp. Morph.* 30 119–141. 4729943

[B10] Gucev Z. Tasic V. Plaseska-Karanfilska D. Dimishkovska M. Laban N. Bozinovski Z. (2019). Severe digital malformations in a rare variant of fibrodysplasia ossificans progressiva. *Am. J. Med. Genet. A* 179 1310–1314. 10.1002/ajmg.a.61153 31012264

[B11] Harrison R. J. Pitcher J. D. Mizel M. S. Temple H. T. Scully S. P. (2005). The radiographic morphology of foot deformities in patients with fibrodysplasia ossificans progressiva. *Foot Ankle Int.* 26 937–941. 10.1177/107110070502601107 16309607

[B12] Huang B.-L. Trofka A. Furusawa A. Norrie J. L. Rabinowitz A. H. Vokes S. A. (2016). An interdigit signalling centre instructs coordinate phalanx-joint formation governed by 5′Hoxd–Gli3 antagonism. *Nat. Commun.* 7:12903. 10.1038/ncomms12903 27713395 PMC5059757

[B13] Kaplan F. S. Xu M. Glaser D. L. Collins F. Connor M. Kitterman J. (2008). Early diagnosis of fibrodysplasia ossificans progressiva. *Pediatrics* 121 e1295–e1300. 10.1542/peds.2007-1980 18450872 PMC3502043

[B14] Kaplan F. S. Xu M. Seemann P. Connor J. M. Glaser D. L. Carroll L. (2009). Classic and atypical fibrodysplasia ossificans progressiva (FOP) phenotypes are caused by mutations in the bone morphogenetic protein (BMP) type I receptor ACVR1. *Hum. Mutat.* 30 379–390. 10.1002/humu.20868 19085907 PMC2921861

[B15] Le Minor J.-M. Mousson J.-F. de Mathelin P. Bierry G. (2016). Non-metric variation of the middle phalanges of the human toes (II-V): long/short types and their evolutionary significance. *J. Anatomy* 228 965–974. 10.1111/joa.12462 27031825 PMC5341584

[B16] Low K. Smith J. Lee S. Newbury-Ecob R. (2013). A mother and daughter with a novel phenotype of hand and foot abnormalities and severe pectus excavatum. *Am. J. Med. Genet. A* 161A 2056–2059. 10.1002/ajmg.a.36016 23824731

[B17] Mishima K. Kitoh H. Haga N. Nakashima Y. Kamizono J. Katagiri T. (2014). Radiographic characteristics of the hand and cervical spine in fibrodysplasia ossificans progressiva. *Intractable Rare Dis. Res.* 3 46–51. 10.5582/irdr.2014.01009 25343126 PMC4204539

[B18] Neil M. J. Conacher C. (1984). Bilateral delta phalanx of the proximal phalanges of the great toes. a report on an affected family. *J. Bone Joint Surg. Br.* 66 77–80. 10.1302/0301-620x.66b1.6693482 6693482

[B19] Nix S. Smith M. Vicenzino B. (2010). Prevalence of hallux valgus in the general population: a systematic review and meta-analysis. *J. Foot Ankle Res.* 3:21. 10.1186/1757-1146-3-21 20868524 PMC2955707

[B20] Pignolo R. J. Baujat G. Brown M. A. De Cunto C. Di Rocco M. Hsiao E. C. (2019). Natural history of fibrodysplasia ossificans progressiva: cross-sectional analysis of annotated baseline phenotypes. *Orphanet J. Rare Dis.* 14:98. 10.1186/s13023-019-1068-7 31053156 PMC6499994

[B21] Raspopovic J. Marcon L. Russo L. Sharpe J. (2014). Digit patterning is controlled by a Bmp-Sox9-Wnt turing network modulated by morphogen gradients. *Science* 345 566–570. 10.1126/science.1252960 25082703

[B22] Ray A. Singh P. N. P. Sohaskey M. L. Harland R. M. Bandyopadhyay A. (2015). Precise spatial restriction of BMP signaling is essential for articular cartilage differentiation. *Development* 142 1169–1179. 10.1242/dev.110940 25758226 PMC4360183

[B23] Rosenstirn J. (1918). A contribution to the study of myositis ossificans progressiva. *Ann. Surg.* 68 485–520. 17864014 10.1097/00000658-191811000-00006 PMC1427091

[B24] Sarrafian S. K. (2011). *Sarrafian’s Anatomy of the Foot and Ankle: Descriptive, Topographic, Functional*, 3rd Edn Philadelphia, PA: LWW.

[B25] Schaffer A. A. Kaplan F. S. Tracy M. R. O’Brien M. L. Dormans J. P. Shore E. M. (2005). Developmental anomalies of the cervical spine in patients with fibrodysplasia ossificans progressiva are distinctly different from those in patients with klippel-feil syndrome: clues from the BMP signaling pathway. *Spine* 30 1379–1385. 10.1097/01.brs.0000166619.22832.2c 15959366

[B26] Schroeder H. W. Zasloff M. (1980). The hand and foot malformations in fibrodysplasia ossificans progressiva. *Johns Hopkins Med. J.* 147 73–78. 7412069

[B27] Shen Q. Little S. C. Xu M. Haupt J. Ast C. Katagiri T. (2009). The fibrodysplasia ossificans progressiva R206H ACVR1 mutation activates BMP-independent chondrogenesis and zebrafish embryo ventralization. *J. Clin. Invest.* 119 3462–3472. 10.1172/JCI37412 19855136 PMC2769180

[B28] Shore E. M. Kaplan F. S. (2010). Inherited human diseases of heterotopic bone formation. *Nat. Rev. Rheumatol.* 6 518–527. 10.1038/nrrheum.2010.122 20703219 PMC3551620

[B29] Shore E. M. Xu M. Feldman G. J. Fenstermacher D. A. Cho T. J. Choi I. H. (2006). A recurrent mutation in the BMP type I receptor ACVR1 causes inherited and sporadic fibrodysplasia ossificans progressiva. *Nat. Genet.* 38 525–527. 10.1038/ng1783 16642017

[B30] Towler O. W. Shore E. M. Kaplan F. S. (2019). Skeletal malformations and developmental arthropathy in individuals who have fibrodysplasia ossificans progressiva. *Bone* 23:115116. 10.1016/j.bone.2019.115116 31655222

[B31] Verma V. Batra A. Singla R. Gogna P. Magu N. Gupta R. (2014). Longitudinal bracketed epiphysis of proximal phalanx of the great toe with congenital hallux varus managed simultaneously with monorail external fixator: a case report. *Foot Ankle Special.* 7 68–70. 10.1177/1938640013502724 24026085

