# Supplementary figures and images for: The Developmental Phenotype of the Great Toe in Fibrodysplasia Ossificans Progressiva
Source: Front Cell Dev Biol. 2020 Dec 8;8:612853. doi: 10.3389/fcell.2020.612853 (PMC7753048; doi:10.3389/fcell.2020.612853)

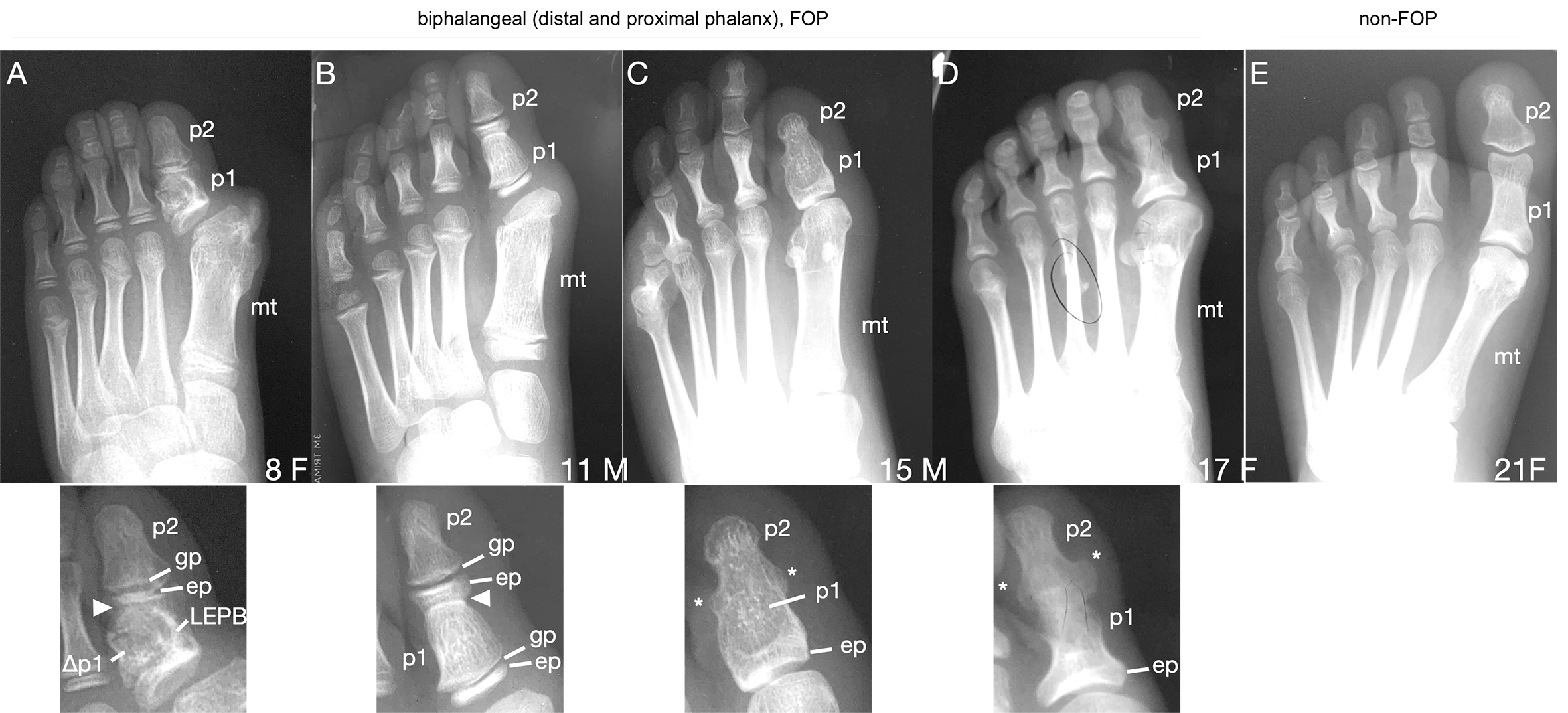

Supplement: Supplementary Figure 1 — Symphalangism of biphalangeal hallux in subjects with FOP. (A–D) Radiographs of four patients at various ages with biphalangeal hallux. (A,B) Subjects with both proximal and distal phalanges (p1, p2) appear to show separation of those phalanges throughout childhood and early adolescence. The phalanges are closely juxtaposed prior to age 15 and may already be fused (higher magnification, below), but show a clear demarcation between the two skeletal elements (arrowheads). (C,D) At ages 15 and older, proximal and distal phalanges are fused. The distinct shapes of the proximal extremities of the phalanges (asterisks) suggest that both phalanges were initially present although could not be verified in subjects lacking radiographs from younger ages. (E) An unaffected patient with clear separation of the proximal and distal phalanges of digit 1. ep, epiphysis; gp, growth plate; LEPB, lateral epiphyseal bracket; mt, metatarsal. Age (in years) and sex (F, M) of each subject, bottom right of each panel. [file Image_1.TIF]
